# Genome-wide identification and characterization of the BES/BZR gene family in wheat and foxtail millet

**DOI:** 10.1186/s12864-021-08002-5

**Published:** 2021-09-21

**Authors:** Dan Liu, Yanjiao Cui, Zilong Zhao, Suying Li, Dan Liang, Conglei Wang, Gang Feng, Jianhe Wang, Zhengli Liu

**Affiliations:** 1 Tianjin Key Laboratory of Crop Genetics and Breeding, Institute of Crop Sciences, Tianjin Academy of Agricultural Sciences, Tianjin, China; 2 Department of Life Sciences, Tangshan Normal University, Tangshan, China

**Keywords:** Brassinosteroid, BES/BZR transcription factor, Environmental stress, Wheat, Foxtail millet

## Abstract

**Background:**

BES/BZR family genes have vital roles in plant growth, development, and adaptation to environmental stimuli. However, they have not yet been characterized and systematically analyzed in wheat and foxtail millet.

**Results:**

In the current study, five common and two unique *BES/BZR* genes were identified by genome-wide analysis in wheat and foxtail millet, respectively. These genes were unevenly distributed on 14 and five chromosomes of wheat and foxtail millet, respectively, and clustered in two subgroups in a phylogenetic analysis. The BES/BZR gene family members in each subgroup contained similar conserved motifs. Investigation of *cis*-acting elements and expression profile analysis revealed that the *BES/BZR* genes were predominantly expressed in leaf tissues of wheat and foxtail millet seedlings and responded to brassinosteroid, abscisic acid, and NaCl treatments.

**Conclusions:**

Our results provide a basis for future studies on the function and molecular mechanisms of the BES/BZR gene family in wheat, foxtail millet, and other plants.

**Supplementary Information:**

The online version contains supplementary material available at 10.1186/s12864-021-08002-5.

## Background

Plant growth and development are coordinately regulated by various phytohormones, including auxin, ethylene (ETH), cytokinin (CTK), gibberellin (GA), and abscisic acid (ABA). Brassinosteroids (BRs), as the sixth plant hormone, are a group of steroidal compounds with important and varied roles in plant physiological processes, agronomic traits, and responses to diverse abiotic and biotic stresses. Deficiencies of BR biosynthesis or signaling lead to distinct growth defects in plants, such as dwarfism, delayed flowering time, premature senescence, and male infertility [1, 2]. Conversely, elevated endogenous BR levels and sensitivity result in increased crop yield and enhanced tolerance to stress [3, 4].

The BR signal transduction pathway has been extensively studied and characterized in the past two decades [1, 2]. The BR signal is perceived at the cell surface by the Leucine-Rich Repeat (LRR) receptor-like kinase, BRASSINOSTEROID INSENSITIVE 1 (BRI1), and its co-receptor BRI1-ASSOCIATED RECEPTOR KINASE 1 (BAK1), which were localized in the plasma membrane [5–8]. After BR recognition, two homologous transcription factors, BRASSINAZOLE RESISTANT 1 (BZR1) and BRI1-EMS-SUPPRESSOR 1 (BES1)/BZR2, are activated and translocated to the nucleus to initiate transcription of downstream target genes by binding directly to BR response elements (BRREs, CGTGT/CG) and E-box elements (CANNTG) in their promoters [9, 10].

The two major transcription factors in BR signaling, BZR1 and BES1, contain a bipartite nuclear localization signal, a plant-specific and highly conserved amino-terminal domain, 22–24 phosphorylation sites, a PEST (rich in proline [P], glutamic acid [E], serine [S], and threonine [T]) motif involved in protein degradation, and a carboxyl-terminal domain [9, 11]. In addition to acting as positive regulators of BR signaling, BZR1 and BES1 mediate crosstalk between BR and diverse signals such as other phytohormones, light, and stress, thereby regulating plant development and environment adaptability. There are various examples of this crosstalk. Firstly, BZR1/BES1 can directly bind to the promoters of several GA biosynthetic genes and control their expression in Arabidopsis and rice (*Oryza sativa* L.) [12, 13]. Secondly, the master negative regulators of GA signaling, DELLAs (SLENDER-RICE1 [SLR1] in rice and GA INSENSITIVE [GAI], REPRESSOR OF GAI-3 [RGA], RGA-LIKE1 [RGL1], RGL2 and RGL3 in Arabidopsis), can inactivate the transcriptional regulatory activity of BZR1 and destabilize the BZR1 protein [14, 15]. Thirdly, increased BES1 levels enhance DNA binding of auxin-regulated transcription factor AUXIN RESPONSIVE FACTOR 5 (ARF5) to its target promoters, and BES1/BZR1 HOMOLOG 4 (BEH4), which is a paralog of BES1, acts in partial redundancy with BES1 for the normal auxin response during growth of Arabidopsis seedlings [16]. A fourth example of the crosstalk is that the transcription factor ZmBES1/BZR1-5 from maize (*Zea mays* L.) decreases sensitivity to ABA and positively regulates salt and drought tolerance in transgenic Arabidopsis [17]. Furthermore, in response to pathogen infection or insect feeding, BES1 inhibits the jasmonate (JA)-inducible transcription of defensin genes *PLANT DEFENSIN 1.2a* (*PDF1.2a*) and *PDF1.2b* and biosynthesis of the JA-induced insect defense-related metabolite indolic glucosinolate, thus demonstrating the antagonism of BRs to JA-activated plant defense responses in Arabidopsis [18]. Finally, BES1 can also interact with D53-like SUPPRESSOR OF MAX2-1-LIKE proteins (SMXLs), which are substrates of strigolactone receptor complex D14-MAX2, and directly inhibit expression of the gene *BRANCHED 1* (*BRC1*) that acts as a key switch for inhibiting shoot branching in Arabidopsis [19].

The pivotal role of BZR1 and BES1 in various biological and developmental processes, as well as in stress responses, has resulted in the identification of the BES/BZR gene family in plants and their regulation mechanisms becoming a research hotspot in recent years. However, to date, the BES/BZR transcription factor family has only been identified and characterized in a limited number of plant species, such as *Arabidopsis thaliana* [9, 20], maize [21, 22], sorghum (*Sorghum bicolor* L.) [23], and some horticultural plants [24–30].

Wheat (*Triticum aestivum* L.) is grown worldwide and is one of the three leading cereal crops globally. Abiotic stresses, such as high salinity and drought, are major environmental conditions that adversely affect wheat growth and severely reduce productivity [31]. Therefore, identifying BES/BZR gene family members in wheat and investigating their biological functions are crucial to improve tolerance of this crop against abiotic stress and raise production. Foxtail millet [*Setaria italica* (L.) P. Beauv.] is a traditional cereal food crop in China and is predominantly cultivated in developing countries as a minor crop [32]. Foxtail millet has strong tolerance to drought and barren soil; consequently, characterization and analysis of BES/BZR family members might help elucidate the underlying molecular mechanism of resistance to stresses in this crop. Moreover, the availability of the whole genome sequence of foxtail millet offers the possibility of genome-wide analysis of BES/BZR gene family members [33, 34]. In addition, comparative analysis on structure, expression pattern and evolution of *BES/BZR* genes in foxtail millet with those in wheat will help elucidate the function of BES/BZR gene family and provide valuable resources for improving of adaptaion to diverse environment stresses in wheat by inhibition or overexpression of these genes.

In the current study, 20 BES/BZR gene family members were identified in wheat and seven were identified in foxtail millet, and their gene location, phylogenetic relationships, conserved protein motif structures, and *cis*-acting regulatory elements in promoters were analyzed. Furthermore, the expression patterns of *BES/BZR* genes in different tissues and in response to phytohormones and stress treatments were determined, and the evolutionary origin of the BES/BZR gene family in wheat and foxtail millet was explored. Findings from the study suggest that the BES/BZR family in wheat and foxtail millet play roles in the BR and ABA signaling pathways and salt stress response. The study provides valuable resources for future work on the function and molecular mechanisms of the BES/BZR family in wheat, foxtail millet, and other plants.

## Methods

### Plant materials

Foxtail millet conventional variety, Jigu32 (abbreviated as JG32), which was preserved by Tangshan Normal University, and wheat variety, C6002, which was provided by Institute of Crop Sciences, Tianjin Academy of Agricultural Sciences, were used in this study. All these materials were planted in the greenhouse of Tangshan Normal University, Tangshan, China. The leaves and roots of 14-day-old seedlings cultured in water were sampled for determination of the tissue-specific expression patterns of *BES/BZR* genes. The leaves of 14-day-old seedlings cultivated in soil were sprayed with 5 µM BR solution, 50 µM ABA solution, or 50 mM NaCl solution, and harvested at 0 (control), 1, 2, 3, 4, 5, and 6 h after treatment to examine the response of *BES/BZR* genes to BR, ABA, and salt stress. Collected materials were frozen in liquid nitrogen and stored at -80 °C for total RNA extraction. Three biological replicates were performed for each sample and each treatment.

### Identification of ***BES/BZR*** genes

The Hidden Markov Model (HMM) file corresponding to the BES/BZR domain was downloaded from the Pfam database (http://pfam.xfam.org/) by using Pfam ID PF05687. HMMER software [35] was used to search the genome data of wheat, foxtail millet, *Physcomitrella patens*, and *Selaginella moellendorffii* for *BES/BZR* genes with an E-value cutoff of 1e^− 5^. One of multiple transcript IDs was retained as representative of the candidate gene, and the existence of conserved BES/BZR domains was further confirmed using the databases NCBI-Conserved Domain Data (CDD) (https://www.ncbi.nlm.nih.gov/Structure/bwrpsb/bwrpsb.cgi), SMART (http://smart.embl-heidelberg.de/), and Pfam. Remaining sequences with no stop codons were removed.

### Phylogenetic analysis and classification

To analyze the evolutionary relationships and origins of BES/BZR transcription factors derived from wheat and foxtail millet, the full-length amino acid sequences of these transcription factors were aligned by ClustalX software with the default parameters, and a neighbor-joining (NJ) phylogenetic tree was reconstructed by using MEGA 7.0 software [36] with bootstrap replications of 1000. The BES/BZR transcription factors were classified into different groups according to the topology of phylogenetic tree.

To study the expression characteristics of *BES/BZR* genes in wheat and foxtail millet, sequences 1500 bp upstream of the start codon (ATG) in the *BES/BZR* genes were acquired and considered as the promoter region, and a phylogenetic tree based on the promoter sequences was reconstructed by using MEGA 7.0 software with bootstrap replications of 1000.

### Conserved motif analysis of BES/BZR proteins

Conserved motifs in the full-length amino acid sequences of BES/BZR proteins in wheat and foxtail millet were predicted by CDD (https://www.ncbi.nlm.nih.gov/Structure/bwrpsb/bwrpsb.cgi, E-value < 1e^− 5^), SMART (http://smart.embl-heidelberg.de/, default parameters), and Pfam (E-value < 1e^− 5^, HMM length > 150), and the motifs shared by these three databases were retained. Finally, the conserved motifs of BES/BZR proteins were exihibited through TBtools software [37].

### Chromosome location and gene duplication

Chromosome positional data of *BES/BZR* genes were retrieved from Ensembl Plants (http://plants.ensembl.org/index.html). Mapping of *BES/BZR* genes was achieved using MapChart software [38].

Gene duplication events were analyzed using the Multiple Collinearity Scan toolkit (MCScanX) [39], and TBtools software was used to generate the syntonic map. For synteny analysis, both the alignment file of protein sequences (BLAST format) and annotation file in the GFF file format of wheat and foxtail millet were extracted and merged. Then the collinear blocks were detected through Quick Run MCScanX package of TBtools, and the intraspecies and interspecies synteny relationships of *BES/BZR* genes in wheat and foxtail millet were constructed and visualized through Dual Synteny Plot package ot TBtools.

### Analysis of ***cis***-acting regulatory elements

The *cis*-acting regulatory elements in the promoter region of *BES/BZR* genes were predicted by using PlantCARE database (http://bioinformatics.psb.ugent.be/webtools/plantcare/html/).

### GO annotation

The GO analysis of *BES/BZR* genes in wheat and foxtail millet was performed using the Blast2GO software and then a free online platform, OmicShare tools (http://www.omicshare.com/tools), was used for data analysis.

### Quantitative real-time PCR analysis

Total RNA extraction and cDNA synthesis were performed as described previously [40]. qRT-PCR utilized SuperReal PreMix Plus (SYBR Green, TIANGEN, China) on a QuantStudio 6 Flex Real-Time PCR system (Applied Biosystems, USA) following the manufacturer’s instructions. The PCR thermal profile comprised an initial denaturation at 95 °C for 15 s, followed by 40 cycles of 95 °C for 10 s and 60 °C for 31 s. The relative expression level of each *BES/BZR* gene was then calculated based on the Eq. 2^−ΔΔCT^ [41]. All data were generated from averages of three independent replicates. The statistical significance of the measured gene expression data was assessed using Student’s *t*-test. All primers are listed in Additional file 1: Table S1.

### Cluster analysis of gene expression data

A cluster analysis was performed by TBtools [37] based on the expression data of *BES/BZR* genes in different tissues of wheat and foxtail millet seedlings, and in leaves of the seedlings treated with BR, ABA, or salt stress.

## Results

### Identification of ***BES/BZR*** genes in wheat and foxtail millet

An HMM search was conducted to identify *BES/BZR* candidates, and the HMM profile of BES/BZR (PF05687) was downloaded from the Pfam protein database. Non-representative transcripts were removed, then the remaining sequences were checked for the BES/BZR domain by using CDD, SMART, and Pfam databases, and incomplete sequences without stop codons were excluded. Seven genes in wheat (20 members in the BB, AA, and DD genomes) and seven genes in foxtail millet were confirmed as BES/BZR gene family members.

### Phylogenetic classification and conserved motif analysis of BES/BZR transcription factors in wheat and foxtail millet

**Fig. 1:**
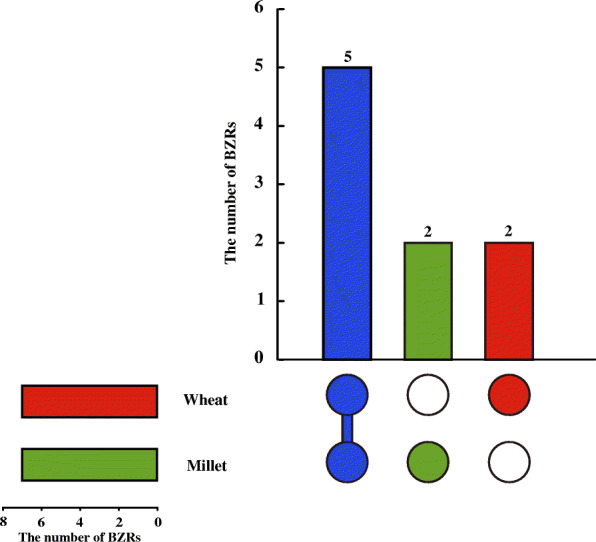
Distribution of BES/BZR proteins in wheat and foxtail millet. The blue column represents the number of common BES/BZR members in wheat and foxtail millet, and the red and green columns represent the number of unique BES/BZR members in wheat and foxtail millet, respectively

Evolutionary relationships among the BES/BZR transcription factors in wheat and foxtail millet were analyzed via an unrooted phylogenetic tree based on their full-length amino acid sequences, and these BES/BZR members were named accordingly. Among the proteins identified in wheat, TaBZR1 was closely related to BZR1 in Arabidopsis [11], TaBZR2-A was defined by Cui et al. [42], and TaBZR3 to TaBZR7 were designated on the basis of their phylogenetic relationships with TaBZR1 and TaBZR2. Names of the seven BES/BZR proteins in foxtail millet were assigned according to their phylogenetic relationships with BES/BZR proteins in wheat. Five BES/BZR proteins identified in this study (BZR1, BZR2, BZR4, BZR6, BRZ7) were common to both wheat and foxtail millet, two (BZR3, BZR5) were unique in wheat, and two (BZR8, BZR9) were only present in foxtail millet (Fig. 1).

**Fig. 2:**
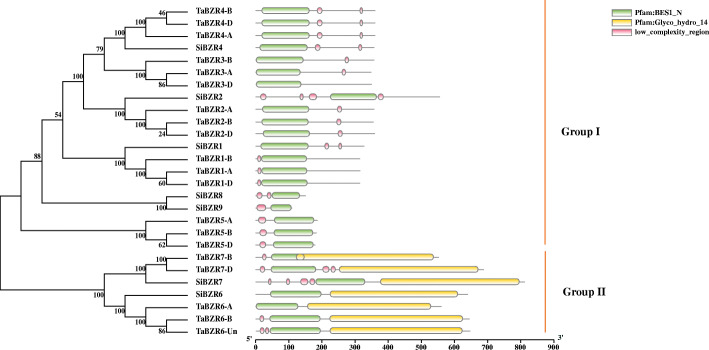
Phylogenetic classification and motif analysis of BES/BZR gene family members from wheat and foxtail millet. Left panel: Unrooted phylogenetic tree reconstructed based on full-length amino acid sequences of BES/BZR proteins in wheat and foxtail millet using MEGA version 7.0 with the neighbor-joining method (bootstrap value = 1000). The BES/BZR proteins were classified into two groups (group I and group II). Right panel: Distribution of conserved motifs for BES/BZR proteins in wheat and foxtail millet. Boxes in different colors represent the motifs

Phylogenetic analysis revealed that the BES/BZR family members in wheat and foxtail millet were divided into two groups (Fig. 2). Fifteen wheat BES/BZR proteins (TaBZR1, TaBZR2, TaBZR3, TaBZR4, and TaBZR5 in the BB, AA, and DD genomes) and five foxtail millet BES/BZRs (SiBZR1, SiBZR2, SiBZR4, SiBZR8, and SiBZR9) belonged to group I, while the remaining five BES/BZR proteins in wheat (TaBZR6-A, TaBZR6-B, TaBZR6-Un, TaBZR7-BB and TaBZR7-D) and two BES/BZR proteins in foxtail millet (SiBZR6 and SiBZR7) clustered in group II.

To explore the structural features of BES/BZR proteins, conserved motifs of the BES/BZR family in wheat and foxtail millet were identified by TBtools software [37] based on their full-length amino acid sequences (Fig. 2 and Additional file 2: Figure S1). Motif analysis showed that all 27 BES/BZR proteins shared one distinguished motif, BES1_N, which was located near the N terminus, the C terminus, or in the middle of these BES/BZR proteins. BES1_N is considered as the most conserved functional motif of BES/BZR family based on its specific binding to the BR-responsive elements and E-box sequences present in many BR-regulated promoters [9, 10, 20, 43]. Another distinguished motif, glycol_hydro_14 that belongs to the glycoside hydrolase family [28, 30], was present in the C-terminal region of the BES/BZR members in group II. Moreover, most of the BES/BZR family members in wheat and foxtail millet, except for TaBZR3-D, TaBZR6-A, and SiBZR6, contained one or more low-complexity regions. The motif analysis demonstrated that the BES/BZR transcription factors in each group shared similar motif features, which suggested that they may have similar functions, and further supported the reliability of the phylogenetic classification of the BES/BZR family members.

### Chromosome location and synteny analysis of ***BES/BZR*** genes in wheat and foxtail millet

In wheat, the seven *TaBES/BZR* genes were mapped randomly on 14 chromosomes and chrUn (Additional file 3: Figure S2). The number of *BES/BZR* members on each chromosome varied from one to five. Chromosomes 6 A and 6B possessed the largest number of *BES/BZR* genes, while chromosomes 2 A, 2B, 2D, 4B, 4D, 6D, 7A, 7B, and 7D only contained a single *BES/BZR* gene. Most *TaBES/BZR* genes were located on the proximate or distal ends of wheat chromosomes. Similarly, the distribution of the seven foxtail millet *BES/BZR* genes was also uneven on five chromosomes. *SiBZR1* and *SiBZR7* were located on chromosome II, *SiBZR8* and *SiBZR9* were present on chromosome III, and *SiBZR2*, *SiBZR4*, and *SiBZR6* were mapped on chromosome V, IV, and I, respectively. Most *SiBES/BZRs* were located on the ends of chromosomes as observed for the *TaBES/BZR* genes; however, based on the scale on the left of Figure S2, it is evident that the foxtail millet chromosomes were much shorter than the wheat chromosomes.

**Fig. 3:**
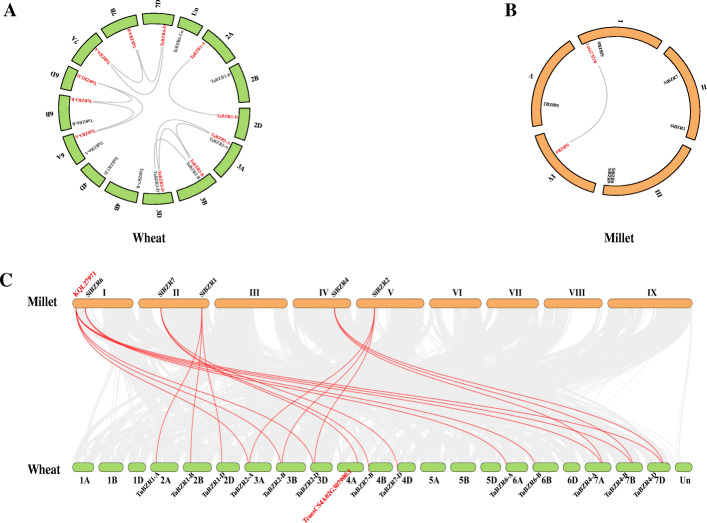
Synteny analysis of the BES/BZR gene family in wheat and foxtail millet. Duplication events occurring in the BES/BZR gene family of wheat (**A**) and foxtail millet (**B**) are represented by gray lines linking *BES/BZR* genes. Syntenic *BES/BZR* gene pairs between wheat and foxtail millet (**C**) are highlighted by red lines. Chromosomes in wheat and foxtail millet are green and orange, respectively

To further investigate the phylogenetic mechanisms of the BES/BZR gene family, a synteny analysis on *BES/BZR* genes in wheat and foxtail millet was conducted (Fig. 3 and Additional file 4: Table S2). A total of 11 duplication events with five *TaBZR* genes (*TaBZR1-A*, *TaBZR1-D*, *TaBZR2-B*, *TaBZR2-D*, *TaBZR3-A*, *TaBZR3-B*, *TaBZR3-D*, *TaBZR4-A*, *TaBZR4-B*, *TaBZR4-D*, *TaBZR5-A*, *TaBZR5-B*, and *TaBZR5-D*) were observed in wheat (Fig. 3 A). In foxtail millet, only chromosomes I and IV (*KQL27971* and *SiBZR4*) showed a syntenic relationship (Fig. 3B). In addition, a comparative syntenic map of wheat associated with foxtail millet was constructed and revealed that the number of orthologous pairs between wheat and foxtail millet was 20 (Fig. 3 C). These results suggested that the BES/BZR syntenic orthologs may have evolved from segment duplication, and that duplication events contributed to the evolution of BES/BZR gene family members from wheat and foxtail millet.

### Phylogenetic classification and analysis of ***cis***-acting elements of ***BES/BZR*** genes in wheat and foxtail millet

**Fig. 4:**
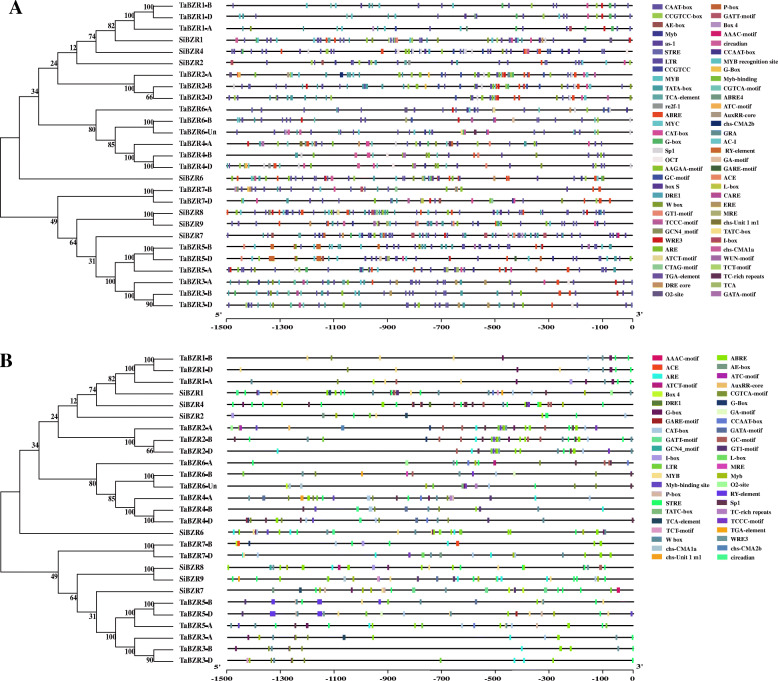
Phylogenetic analysis and *cis*-acting elements analysis in the promoter regions of *BES/BZR* genes from wheat and foxtail millet. All identified *cis*-acting elements and those related to responses to phytohormone and environmental stimuli were shown in (**A**) and (**B**) with phylogenetic analysis, respectively. Left panel: Unrooted phylogenetic tree reconstructed on the basis of promoter sequences of *BES/BZR* genes in wheat and foxtail millet using MEGA version 7.0 with the neighbor-joining method (bootstrap value = 1000). Right panel: Distribution of *cis*-acting elements in promoter regions of *BES/BZR* genes from wheat and foxtail millet. Boxes in different colors represent the *cis*-regulatory elements. Scale bar at the bottom indicates length of promoter sequence

The expression characteristics of *BES/BZR* genes in wheat and foxtail millet were studied by extracting approximately 1500-bp sequences upstream of the coding sequences of *BES/BZR* genes as promoter regions, and performing phylogenetic analysis of these promoter sequences. BES/BZR gene family members in wheat and foxtail millet were classified into two groups based on their promoter sequences (Fig. 4). Membership of these groups differed from that of the groups classified on the basis of full-length amino acid sequences (Fig. 2). In the promoter region classification, group I contained four *BES/BZR* genes in wheat (*TaBZR1*, *TaBZR2*, *TaBZR4*, and *TaBZR6*) and four genes in foxtail millet (*SiBZR1*, *SiBZR2*, *SiBZR4*, and *SiBZR6*), while group II included three genes in wheat (*TaBZR3*, *TaBZR5*, and *TaBZR*7) and three genes in foxtail millet (*SiBZR7*, *SiBZR8*, and *SiBZR9*). Further analysis of the *cis*-acting elements in the promoter regions of *BES/BZR* genes using PlantCARE revealed that a total of 66 *cis*-acting elements were present in the promoters of *BES/BZR* genes, and these elements were related to different functions (Fig. 4, Additional file 5: Figure S3, Additional file 6: Table S3). Apart from TATA box and CAAT box, the most abundant *cis*-acting element was phytohormone-responsive element, followed by light-responsive elements, and finally tissue-specific-expression elements and elements related to stress response. The phytohormone-responsive elements included those responding to ABA (ABRE), MeJA (CGTCA-motif), SA (TCA element), GA (P-box and GARE-motif), and auxin (AuxRR core and TGA-elements). The stress-responsive elements included those responding to low-temperature, drought and salt stresses, such as LTR, MYB, DRE1, STRE and TC-rich repeats. These results suggested that BES/BZR family members in wheat and foxtail millet may function in responses to phytohormones and stress. In addition, the numbers of phytohormone-responsive and stress-responsive elements in the promoters of different BES/BZR members varied, and the types of phytohormones and stresses these elements responded to also differed. For example, the promoter of *TaBZR2* included five ABRE, while the *SiBZR2* promoter contained only one ABRE. The promoter of *TaBZR2* possessed *cis*-acting elements responding to ABA, auxin, and MeJA; however, the promoter of *TaBZR1* contained only one MeJA-responsive element. This indicated that the regulatory effects of different *BES/BZR* genes involved in phytohormone and stress response signaling pathways were distinct.

### GO annotation of ***BES/BZR*** genes in wheat and foxtail millet

**Fig. 5:**
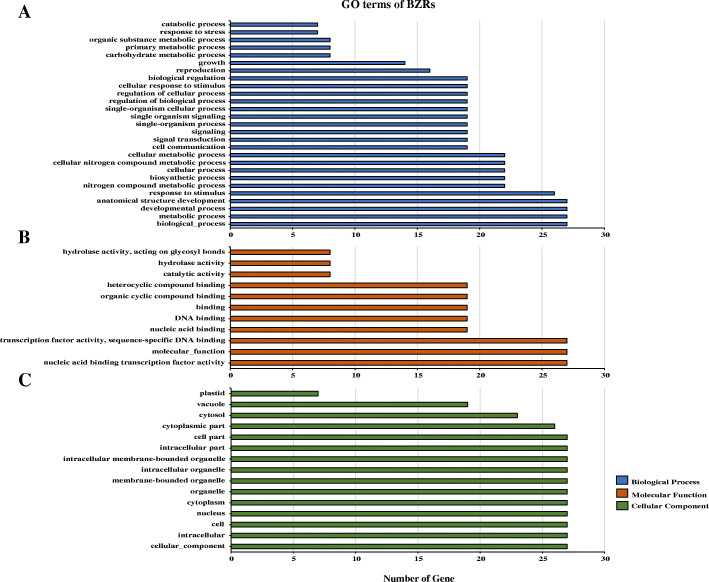
GO annotation of *BES/BZR* genes in wheat and foxtail millet. The annotation was performed on three categories, biological process (**A**), molecular function (**B**) and cellular component (**C**). The GO terms are shown on the Y axis, and the number of genes are shown on the X axis

To further study the biological processes associated with *BES/BZR* genes in wheat and foxtail millet, a GO annotation was performed (Fig. 5 and Additional file 7: Table S4). The biological process analysis showed that *BES/BZR* genes participated in multiple biological processes, and the top five terms were biological process (GO:0008150), metabolic process (GO:0008152), developmental process (GO:0032502), anatomical structure development (GO:0048856) and response to stimulus (GO:0050896). In molecular function section, nucleic acid binding transcription factor activity (GO:0001071), molecular function (GO:0003674) and transcription factor activity, sequence-specific DNA binding (GO:0003700) involved the greatest number of *BES/BZR* genes. The cellular component analysis revealed that besides nucleus, the BES/BZR proteins may also locate on cytoplasm and some intracellular organelles, such as vacuole and plastid. All these results implied that as transcription factors, the BES/BZR family members in wheat and foxtail millet could function in various developmental and metabolic processes, and respond to stress, which provided further support for the results of *cis*-acting elements analysis.

### Tissue-specific expression profiles of ***BES/BZR*** genes in wheat and foxtail millet

**Fig. 6:**
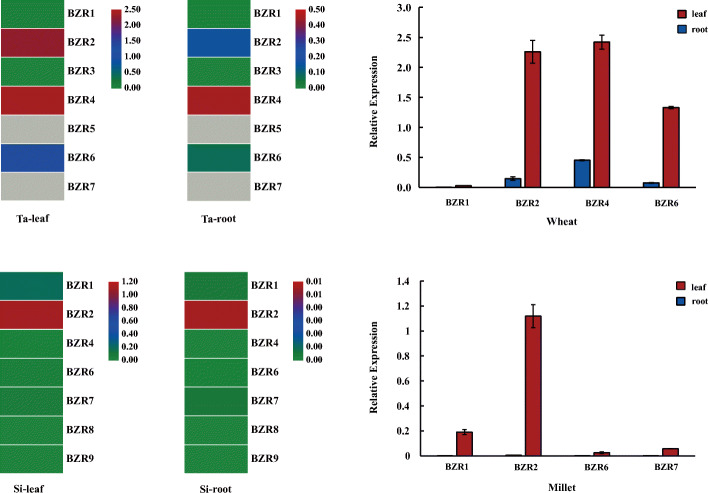
Expression patterns of *BES/BZR* genes in leaf and root tissues of wheat and foxtail millet. Left panel: Heatmaps of expression profiles of different *BES/BZR* genes in leaf and root tissues. Transcript levels are indicated in different colors; red and green represent low and high expression levels, respectively. Right panel: Expression profiles of *BZR1*, *BZR2*, *BZR4*, and *BZR6* in wheat and *BZR1*, *BZR2*, *BZR6*, and *BZR7* in foxtail millet. *ACTIN* genes in wheat and foxtail millet were used as internal controls. Error bars represent standard deviation (SD). Each sample has three biological replicates

To understand the expression patterns of *BES/BZR* genes in different tissues of wheat and foxtail millet, the leaves and roots of 14-day-old seedlings cultured in water were harvested and the transcript levels of different *BZR* genes were analyzed (Fig. 6). In wheat seedlings, expression of *BZR1*, *BZR2*, *BZR4*, and *BZR6* could be detected in roots and leaves. The expression trend was consistent in roots and leaves, with the transcript levels ranking from high to low as *BZR4*, *BZR2*, *BZR6*, and *BZR1*. Moreover, the transcript level of each *BES/BZR* gene in leaves was higher than that in roots. In foxtail millet seedlings, *BZR1*, *BZR2*, *BZR6*, and *BZR7* expression was higher in leaves than in roots, while the expression of *BZR4*, *BZR8*, and *BZR9* could not be measured in 14-day-old seedlings. The order of transcript levels ranked from high to low was *BZR2*, *BZR1*, *BZR7*, and *BZR6*. These expression analyses revealed that *BES/BZR* genes were predominantly expressed in leaf tissues of wheat and foxtail millet seedlings, and that the expression characteristics of *BES/BZR* genes were species-specific. For example, *BZR4* was highly expressed in wheat, whereas the expression of *BZR4* in foxtail millet was rarely detected.

### Expression profiles of ***BES/BZR*** genes under hormones and abiotic stresses

**Fig. 7:**
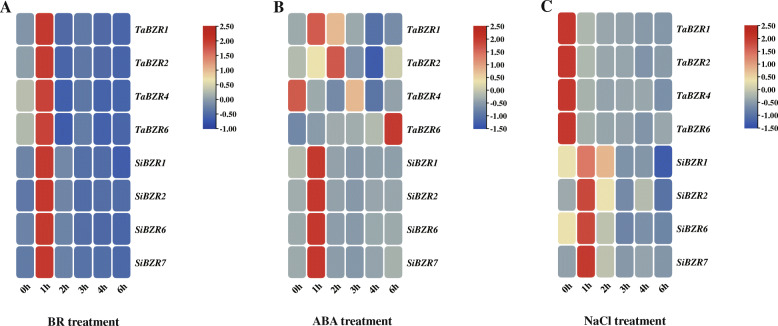
Expression analysis of *BES/BZR* genes in wheat and foxtail millet under hormone and salt stress treatments. The expression profiles of different *BES/BZR* genes under brassinosteroid (BR) (**A**), abscisic acid (ABA) (**B**), and NaCl treatments (**C**) in leaves of wheat and foxtail millet were shown in a heatmap. Transcript levels are indicated in different colors; blue and red represent low and high expression levels, respectively. *ACTIN* genes in wheat and foxtail millet were used as internal controls. Each treatment has three biological replicates

The roles of *BES/BZR* genes in response to plant hormones and abiotic stresses were further investigated by examining the transcript levels of *BES/BZR* genes in leaves under different hormone and stress treatments (Fig. 7, Additional file 8: Figure S4). Expression of *BZR1*, *BZR2*, *BZR4*, and *BZR6* in wheat significantly increased 1 h after treatment with 5 µM BR and then decreased. After spraying with 50 µM ABA, the expression of *BZR1* in wheat was induced at 1 h and then decreased to the control level at 3 h, while the transcript level of *BZR2* remained unchanged over the time course. Expression of *BZR4* was slightly downregulated under ABA treatment, whereas the expression of *BZR6* was markedly increased at 6 h. Moreover, expression of *BZR1*, *BZR2*, *BZR4*, and *BZR6* in wheat was significantly suppressed by 50 mM NaCl at all time points.

Similar to the observations in wheat, the expression of *BZR1*, *BZR2*, *BZR6*, and *BZR7* in foxtail millet was significantly upregulated 1 h after treatment with 5 µM BR. In response to 50 µM ABA, transcript levels of these four *BES/BZR* genes in foxtail millet were also all significantly increased at 1 h, with the transcript levels of *BZR6* and *BZR7* showing a greater increase than those of *BZR1* and *BZR2*. After spraying with 50 mM NaCl, expression of *BZR1*, *BZR2*, *BZR6*, and *BZR7* in foxtail millet was significantly induced at 1 h, with transcript levels of *BZR7* showing a greater increase than those of *BZR1*, *BZR2*, and *BZR6*. The transcript levels of these *BZR* genes then decreased gradually over the remaining time points. These results demonstrated that the *BES/BZR* genes in wheat and foxtail millet were responsive to BR, ABA, and NaCl stresses, and further supported their functions in hormone signaling pathways and the salt stress response. In addition, the response of the *BES/BZR* genes to ABA and NaCl treatment differed between wheat and foxtail millet.

**Fig. 8:**
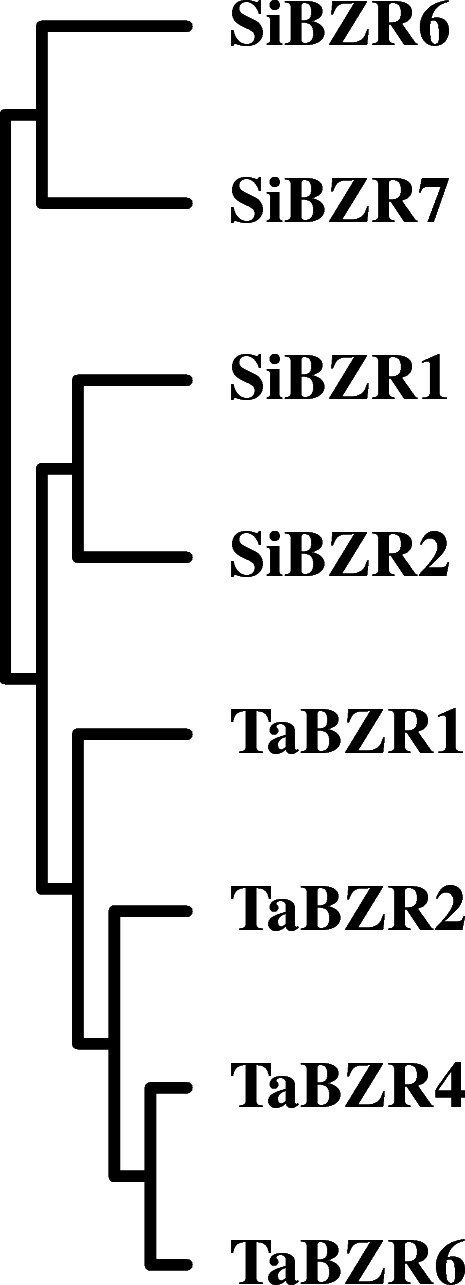
Cluster analysis of *BES/BZR* gene expression data in wheat and foxtail millet seedlings. Data are from expression pattern analysis of *BES/BZR* genes in leaf and root tissues and leaves under BR, ABA and NaCl stress

A cluster analysis based on the expression pattern data of *BES/BZR* genes in leaf and root tissues and under BR, ABA, and NaCl stress was performed using TBtools [37]. The genes *SiBZR1* and *SiBZR2* were grouped in a clade with *TaBZR1*, *TaBZR2*, *TaBZR4*, and *TaBZR6*, while *SiBZR6* and *SiBZR7* formed a separate clade (Fig. 8). These results were basically consistent with the phylogenetic analysis of promoter sequences (Fig. 4), although *SiBZR6* and *SiBZR7* belong to different clades as per their promoter sequence phylogeny, which may be owing to other regulatory elements play roles in gene expression.

### Evolutionary analysis of BES/BZR proteins in plant species

**Fig. 9:**
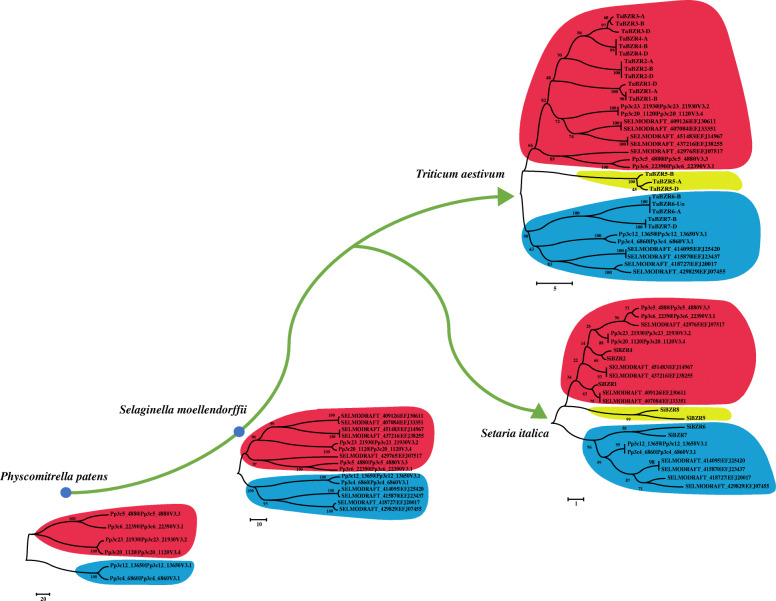
Phylogenetic analysis of BES/BZR gene family members in *Physcomitrella patens*, *Selaginella moellendorffii*, wheat, and foxtail millet. The unrooted phylogenetic tree was reconstructed based on full-length amino acid sequences of BES/BZR proteins using MEGA version 7.0 with the neighbor-joining method (bootstrap value = 1000). Numbers on branches indicate the bootstrap values supporting evolution

To clarify the evolutionary origin of the BES/BZR family in wheat and foxtail millet, nine BES/BZR transcription factors were identified in *S. moellendorffii* and six were identified in *P. paten*, and a phylogenetic analysis was performed based on full-length amino acid sequences of BES/BZR proteins from these two species plus wheat and foxtail millet (Fig. 9). Twelve BES/BZR proteins in wheat (TaBZR1, TaBZR2, TaBZR3, and TaBZR4) were clustered into a clade with four BES/BZR proteins in *S. moellendorffii* (Pp3c23_219030V3.2, Pp3c20_1120V3.4, Pp3c5_4880V3.3, and Pp3c6_22390V3.1) and five BES/BZR proteins in *P. paten* (EFJ30611, EFJ33351, EFJ14967, EFJ38255, and EFJ07517). This suggested that these 12 BES/BZRs in wheat may have originated from the four BZRs in *S. moellendorffii* and five BZRs in *P. paten* Five BZRs in wheat (TaBZR6 and TaBZR7) were classified in a clade with two BZRs in *S. moellendorffii* (Pp3c12_13650V3.1 and Pp3c4_6860V3.1) and four BZRs in *P. paten* (EFJ25420, EFJ23437, EFJ20017, and EFJ07455), suggesting that they may have evolved from a common ancestor. The other three BZRs in wheat (TaBZR5) formed a single clade, indicating that they may have evolved independently.

Among the seven BZRs in foxtail millet, three (SiBZR1, SiBZR2, and SiBZR4) belonged to a clade with four BZRs in *S. moellendorffii* (Pp3c23_219030V3.2, Pp3c20_1120V3.4, Pp3c5_4880V3.3, and Pp3c6_22390V3.1) and five BZRs in *P. paten* (EFJ30611, EFJ33351, EFJ14967, EFJ38255, and EFJ07517), suggesting their common origin. Two BZRs in foxtail millet (SiBZR6 and SiBZR7) grouped in a clade with two BZRs in *S. moellendorffii* (Pp3c12_13650V3.1 and Pp3c4_6860V3.1) and four BZRs in *P. paten* (EFJ25420, EFJ23437, EFJ20017 and EFJ07455). The two remaining BZRs in foxtail millet (SiBZR8 and SiBZR9) formed a separate clade, indicating that they may have evolved independently.

## Discussion

Plants often encounter abiotic and biotic stresses that severely affect growth and cause a decrease in productivity and quality. The BES/BZR proteins are a large family of plant-specific transcription factors. As key components of the BR response pathway, BES/BZR proteins are not only involved in plant growth and developmental processes, but also respond to environmental stresses, such as drought, high salinity, and cold and heat stresses, in a variety of crops including Arabidopsis, *Brassica rapa*, *Brassica napus*, tomato, and maize [17, 18, 25, 28, 44]. Identification and characterization of BES/BZR transcription factors is therefore crucial to understand the molecular mechanism (s) underlying the environmental adaptions of plants to biotic and abiotic stresses. In the past decade, BES/BZR family members have been found in 149 plant species [45]; however, characterization and systematic analysis of BES/BZRs in wheat and foxtail millet has not been completed to date.

In the present study, 20 BES/BZR gene family members were identified in wheat and seven BES/BZR members were identified in foxtail millet. To investigate the evolution and origin of these BES/BZR proteins, a phylogenetic tree was reconstructed based on the full-length amino acid sequences of BES/BZR proteins from some representative plants (Fig. 9). BES/BZR proteins were not identified in algae species, such as *Chlamydomonas reinhardtii*, *Ostreococcus lucimarinus*, and *Galdieria sulphuraria*, but the ancestor of BES/BZRs from wheat and foxtail millet could be found in *P. patens* and *S. moellendorffii*. This suggested that the BES/BZR family only existed in higher plants and that it may play vital roles in the evolution of higher plants, consistent with previous studies [24, 28].

According to the phylogenetic analysis, the *BES/BZR* genes were classified into two groups (Fig. 2), similar to the classification of *BES/BZR* genes observed in maize [22]. The closely related BES/BZRs in group I (TaBZR1, TaBZR2, TaBZR3, TaBZR4, SiBZR1, SiBZR2, and SiBZR4) may have evolved from the four BZRs in *S. moellendorffii* (Pp3c23_219030V3.2, Pp3c20_1120V3.4, Pp3c5_4880V3.3, and Pp3c6_22390V3.1) and the five BZRs in *P. patens* (EFJ30611, EFJ33351, EFJ14967, EFJ38255, and EFJ07517). The BES/BZRs in group II (TaBZR6, TaBZR7, SiBZR6, and SiBZR7) may have common evolutionary origins in *S. moellendorffii* (Pp3c12_13650V3.1 and Pp3c4_6860V3.1) and *P. patens* (EFJ25420, EFJ23437, EFJ20017, and EFJ07455). However, the ancestor of TaBZR5, SiBZR8, and SiBZR9 in group I could not be found in *S. moellendorffii* and *P. patens*, suggesting that these three proteins may have originated after the divergence of seed plants from bryophytes and pteridophytes, later than other BES/BZRs in wheat and foxtail millet. In addition, *BES/BZR* genes belonging to the same group had similar conserved motifs (Fig. 2), suggesting that they exhibit common molecular functions. For example, a BES/BZR transcription factor in wheat, TaBZR2 (TraesCS3A02G139000), was reported to mediate crosstalk between BR and drought signaling pathways, and overexpression of *TaBZR2* enhanced drought tolerance in transgenic wheat plants [42]. Therefore, it is speculated that the BES/BZRs closely related to TaBZR2 may also function in regulation of drought responses in wheat and foxtail millet.

Numerous studies have shown that *BES/BZR* genes respond to various signals, including BR, ABA, indoleacetic acid (IAA), SA, MeJA, GA, NaCl, and polyethylene glycol (PEG) [17, 22, 24, 29, 46]. In the current study, *cis*-acting element analysis revealed that the promoter of *BES/BZR* genes in wheat and foxtail millet contained some phytohormone-responsive elements, including those responding to ABA, MeJA, SA, GA, and auxin, and some stress-responsive elements, such as those responding to low-temperature, drought and high salinity (Fig. 4). This implied that these *BES/BZR* genes are involved in phytohormone and stress response signaling pathways. Subsequently, GO annotation and expression pattern analysis further supported the regulatory effects of the *BES/BZR* genes. Expression of *TaBZR1*, *TaBZR2*, *TaBZR4, TaBZR6*, *SiBZR1*, *SiBZR2*, *SiBZR6i*, and *SiBZR7* could be detected in leaves and roots of 14-day-old seedlings of wheat and foxtail millet, with the highest expression predominantly in leaves (Fig. 6). Following BR treatment, all eight of these genes in wheat and foxtail millet were dramatically upregulated at 1 h (Fig. 7, Additional file 8: Figure S4). After spraying seedlings with ABA, the transcript levels of four *BES/BZR* genes in foxtail millet were significantly induced at 1 h (Fig. 7, Additional file 8: Figure S4). In wheat seedlings sprayed with ABA, *TaBZR1*, and *TaBZR6* were significantly induced at 1 and 6 h, respectively, while transcript levels of *TaBZR2* showed no obvious changes, and the expression of *TaBZR4* was slightly decreased (Fig. 7, Additional file 8: Figure S4). In maize, the *BES/BZR* genes in different tissues were differentially expressed in response to ABA induction [22]. After NaCl treatment, expression of *BES/BZR* genes in wheat was suppressed, while the expression of *BES/BZR* genes in foxtail millet was induced (Fig. 7, Additional file 8: Figure S4). In maize, expression of *BES/BZR* genes was found to be induced and inhibited by NaCl treatment in shoot and root samples, respectively [17]. These results indicated that *BES/BZR* genes in wheat and foxtail millet responded to BR, ABA, and NaCl stresses. Furthermore, *BES/BZR* genes in different plant species and *BES/BZR* family members of the same species could have different responses to phytohormones or environmental stresses, suggesting their functional diversification in hormone signaling pathways and stress responses. BRs and ABA play a pivotal role in promoting environmental adaptions to diverse biotic and abiotic stresses, such as drought, salinity, heat, and cold, and these environmental challenges severely impact plant performance and productivity. Therefore, these *BES/BZR* genes responded to BR, ABA, and NaCl stresses could be served as candidates for crop improvement, and further research on the regulatory effects and mechanisms of *BES/BZR* genes in wheat and foxtail millet is crucial for increasing tolerance to environmental stresses and enhancing crop yield and quality by genetic improvement.

Prediction of *cis*-acting elements in gene promoters can aid the expression profile analysis of genes. Clustering of the *BES/BZR* genes based on their expression profile data (Fig. 8) was basically consistent with the phylogenetic analysis of promoter sequences and *cis*-elements (Fig. 4), but differed from the phylogenetic analysis based on amino acid sequences of *BZR/BES* genes (Fig. 2). This indicated that the expression characteristics of *BZR/BES* genes in wheat and foxtail millet were closely related with their *cis*-elements. For better understanding of the relationship between *cis*-acting regulatory elements in the promoter region and expression data, a Pearson’s correlation coefficient analysis was employed (Additional file 9: Table S5). The results revealed a strong positive relation between the expression data of *BES/BZR* genes in leaves of the seedlings treated with BR and some phytohormone-responsive elements (CGTCA-motif, ABRE and AuxRR core), some light-responsive elements (ACE, chs-Unit1m1, GATA-motif, Sp1, ATC-motif, TCCC-motif and GT1-motif), and several stress-responsive elements (LTR and TC-rich repeats), implying that in addition to their involvement in BR signaling, these BES/BZR proteins may mediate crosstalk between BR and other signals, such as MeJA, auxin, ABA, light and cold. Similarly, the positive correlation between expression data of *BES/BZR* genes in leaves of the seedlings treated with ABA or NaCl and several *cis*-acting elements suggested the participation of BES/BZR proteins in crosstalk between various phytohormone signals and environmental stress signals. However, the biological functions of BES/BZR proteins require further study.

In this study, we found that several *BES/BZR* genes were common to both wheat and foxtail millet, and several genes were unique, such as *BZR3* and *BZR5* in wheat and *BZR8* and *BZR9* in foxtail millet (Fig. 1). Moreover, the expression characteristics of several *BES/BZR* genes were specific. For example, the expression of *BZR4* and *BZR7* were only detected in tissues of wheat and foxtail millet, respectively (Fig. 6). These genes may have roles in controlling important agronomic traits in wheat or foxtail millet, and the elucidation of their function and molecular mechanism underlying will be a focus of the future study.

In conclusion, this study is the first to systematically and comprehensively analyze the BES/BZR family in wheat and foxtail millet. The preliminary exploration of BES/BZR transcription factors provides useful information for future research on the biological functions of these transcription factors, and lays a foundation for the study of the BES/BZR family in other species.

## Supplementary Information

**Additional file 1.**

**Additional file 2.**

**Additional file 3.**

**Additional file 4.**

**Additional file 5.**

**Additional file 6.**

**Additional file 7.**

**Additional file 8.**

**Additional file 9.**

## Data Availability

All data generated or analysed during this study are included in this article and its supplementary material.

## Abbreviations

ETH: Ethylene
CTK: Cytokinin
GA: Gibberellin
ABA: Abscisic acid
BRs: Brassinosteroids
LRR: Leucine-Rich Repeat
BRI1: BRASSINOSTEROID INSENSITIVE 1
BAK1: BRI1-ASSOCIATED RECEPTOR KINASE 1
BZR1: BRASSINAZOLE RESISTANT 1
BES1: BRI1-EMS-SUPPRESSOR 1
BRREs: BR response elements
SLR1: SLENDER-RICE1
GAI: GA INSENSITIVE
RGA: REPRESSOR OF GAI-3
RGL1: RGA-LIKE1
ARF5: AUXIN RESPONSIVE FACTOR 5
BEH4: BES1/BZR1 HOMOLOG 4
PDF1.2a: PLANT DEFENSIN 1.2a
SMXLs: SUPPRESSOR OF MAX2-1-LIKE proteins
BRC1: BRANCHED 1
CDD: NCBI-Conserved Domain Data
MCScanX: Multiple Collinearity Scan toolkit
IAA: Indoleacetic acid
PEG: Polyethylene glycol
