# Supplementary figures and images for: Genome-wide identification and characterization of the BES/BZR gene family in wheat and foxtail millet
Source: BMC Genomics. 2021 Sep 21;22:682. doi: 10.1186/s12864-021-08002-5 (PMC8456565; doi:10.1186/s12864-021-08002-5)

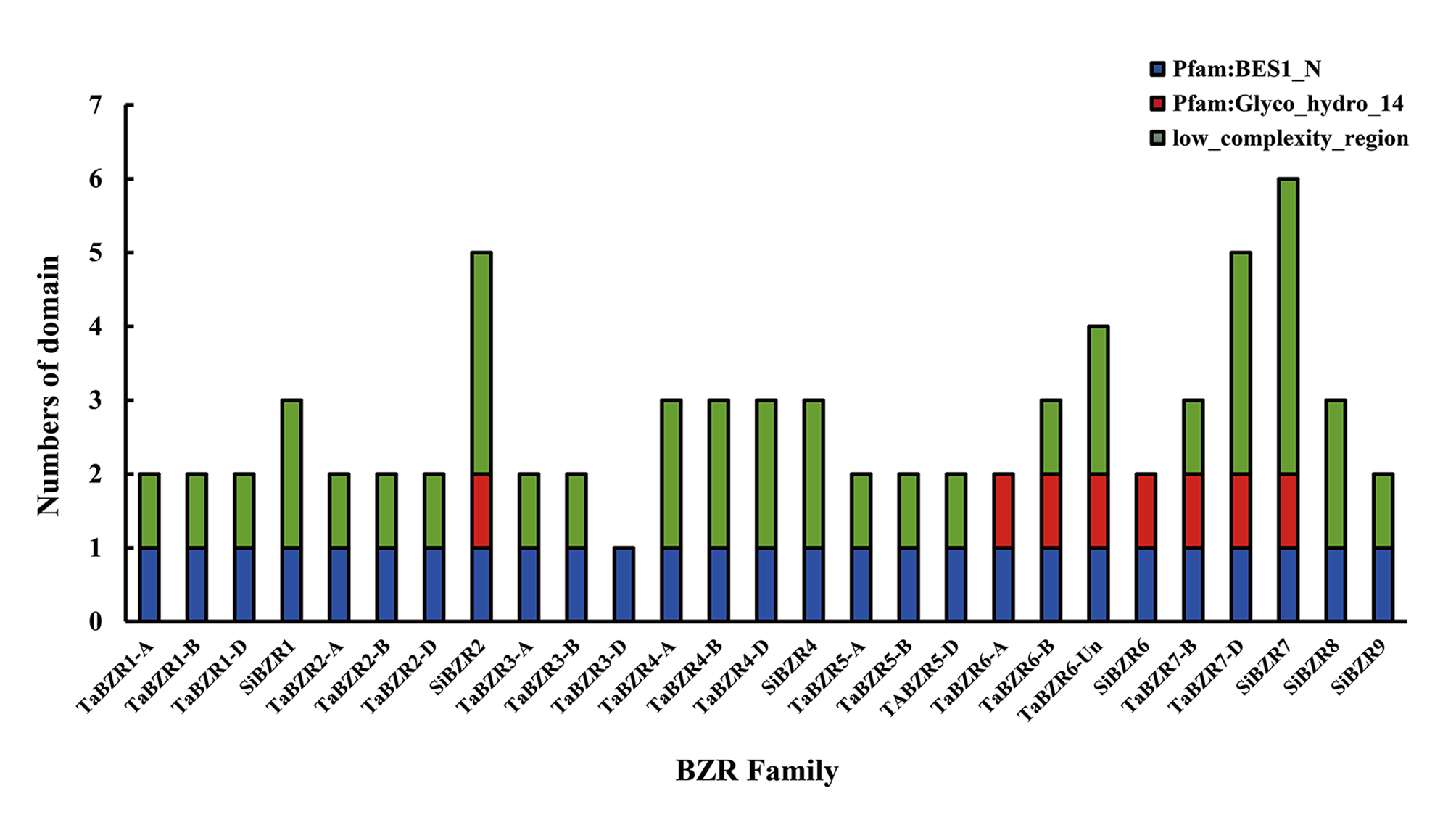

Supplement: Supplementary file 2 — Additional file 2. [file 12864_2021_8002_MOESM2_ESM.tif]

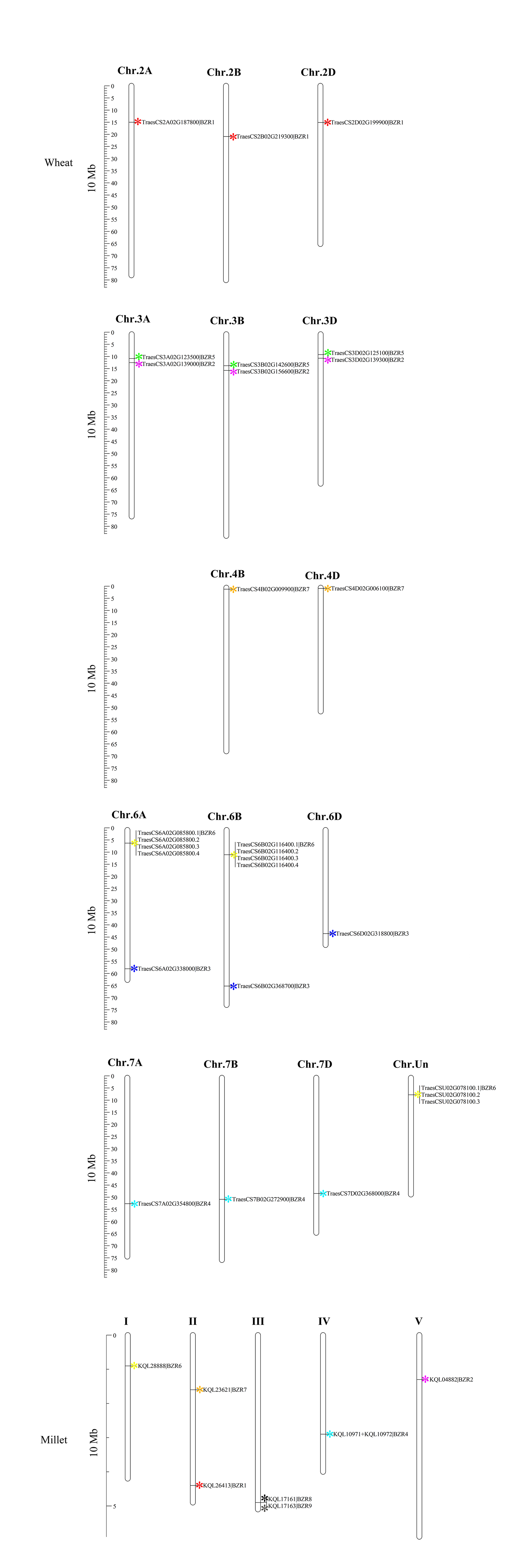

Supplement: Supplementary file 3 — Additional file 3. [file 12864_2021_8002_MOESM3_ESM.tif]

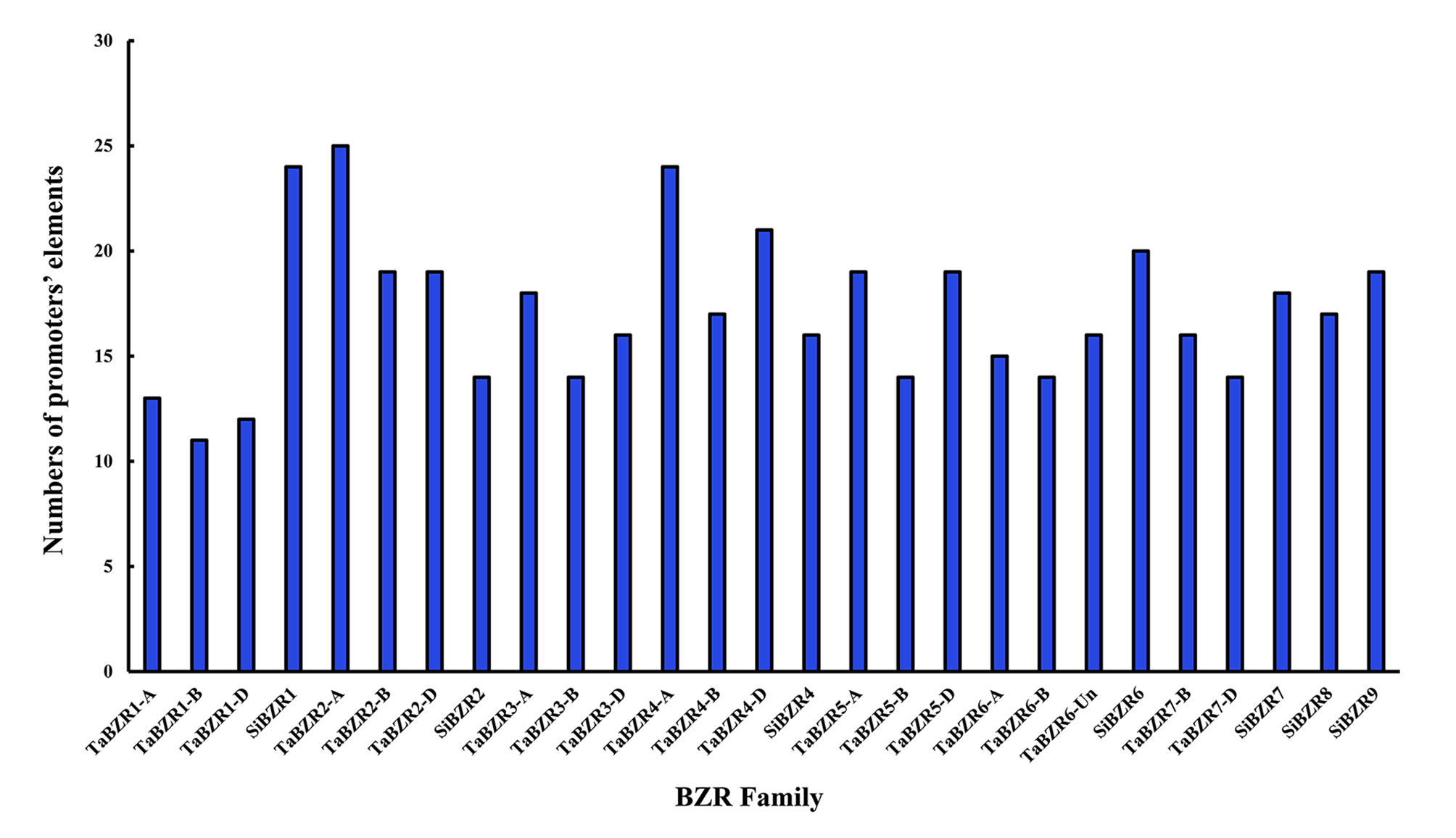

Supplement: Supplementary file 5 — Additional file 5. [file 12864_2021_8002_MOESM5_ESM.tif]

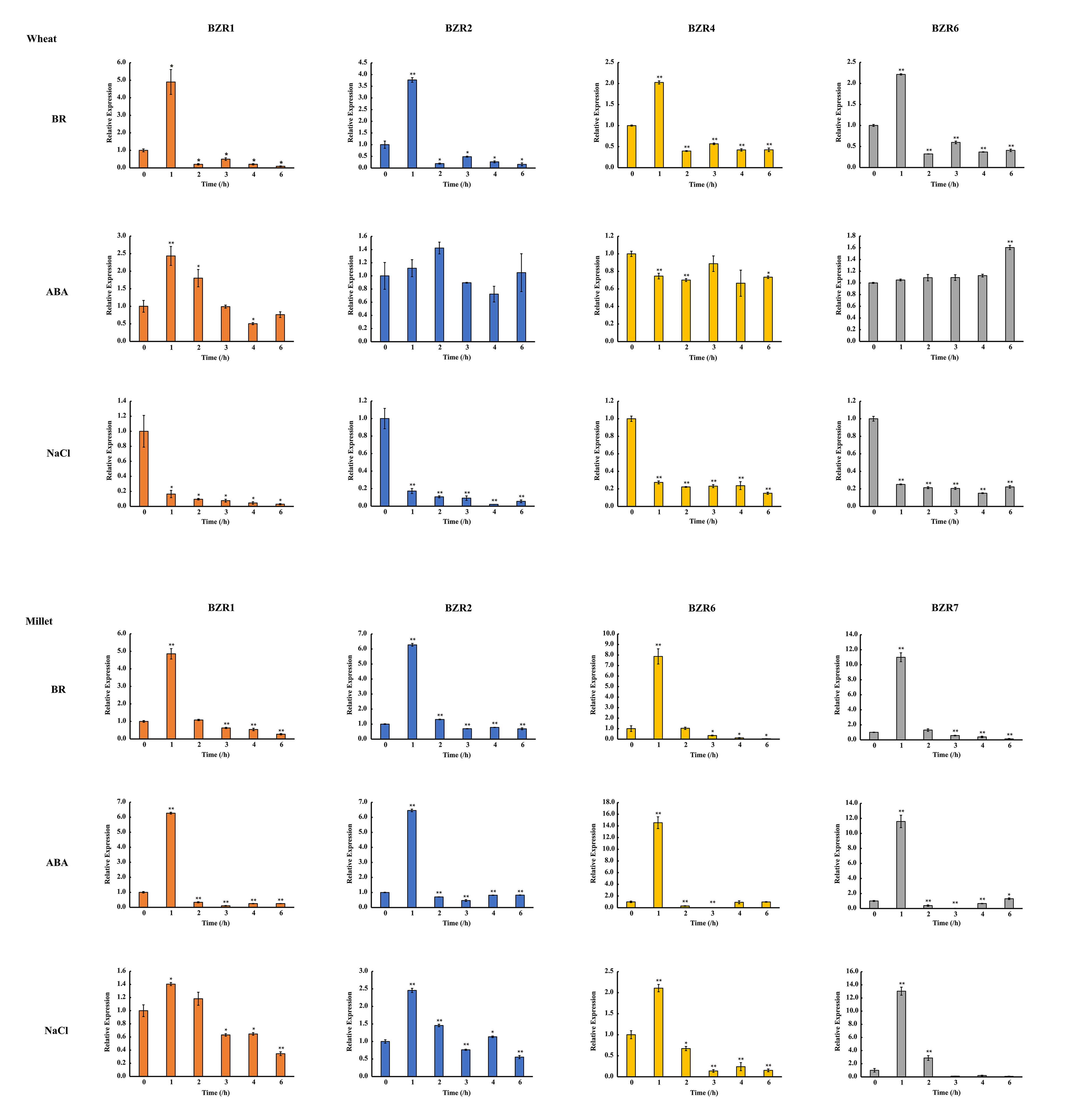

Supplement: Supplementary file 8 — Additional file 8. [file 12864_2021_8002_MOESM8_ESM.tif]
